# Stent treatment or surgical closure for perforated duodenal ulcers: a prospective randomized study

**DOI:** 10.1007/s00464-020-08158-3

**Published:** 2020-11-30

**Authors:** Jorge Alberto Arroyo Vázquez, Kaveh Khodakaram, Maria Bergström, Per-Ola Park

**Affiliations:** 1 Department of Surgery, South Älvsborg Hospital, Brämhultsvägen 53, 501 82 Borås, Sweden; 2 Department of Surgery, Sahlgrenska University Hospital, Gothenburg, Sweden; 3 Department of Surgery, Halland Hospital, Varberg, Sweden; 4 University of Gothenburg, Sahlgrenska Academy, Gothenburg, Sweden

**Keywords:** Perforated duodenal ulcer, Duodenal stent, Peptic ulcer perforation

## Abstract

**Background:**

Perforated peptic ulcer is a life-threatening condition. Traditional treatment is surgery. Esophageal perforations and anastomotic leakages can be treated with endoscopically placed covered stents and drainage. We have treated selected patients with a perforated duodenal ulcer with a partially covered stent. The aim of this study was to compare surgery with stent treatment for perforated duodenal ulcers in a multicenter randomized controlled trial.

**Methods:**

All patients presenting at the ER with abdominal pain, clinical signs of an upper G-I perforation, and free air on CT were approached for inclusion and randomized between surgical closure and stent treatment. Age, ASA score, operation time, complications, and hospital stay were recorded. Laparoscopy was performed in all patients to establish diagnosis. Surgical closure was performed using open or laparoscopic techniques. For stent treatment, a per-operative gastroscopy was performed and a partially covered stent was placed through the scope. Abdominal lavage was performed in all patients, and a drain was placed. All patients received antibiotics and intravenous PPI. Stents were endoscopically removed after 2–3 weeks. Complications were recorded and classified according to Clavien-Dindo (C-D).

**Results:**

43 patients were included, 28 had a verified perforated duodenal ulcer, 15 were randomized to surgery, and 13 to stent. Median age was 77.5 years (23–91) with no difference between groups. ASA score was unevenly distributed between the groups (*p* = 0.069). Operation time was significantly shorter in the stent group, 68 min (48–107) versus 92 min (68–154) (*p* = 0.001). Stents were removed after a median of 21 days (11–37 days) without complications. Six patients in the surgical group had a complication and seven patients in the stent group (C-D 2–5) (n.s.).

**Conclusions:**

Stent treatment together with laparoscopic lavage and drainage offers a safe alternative to traditional surgical closure in perforated duodenal ulcer. A larger sample size would be necessary to show non-inferiority regarding stent treatment.

## Background

Perforated peptic ulcer is a life-threatening complication in ulcer disease. About 2–14% of all peptic ulcers are believed to perforate, with an incidence of 4–11/100,000 per year in northern Europe [1]. One third of all perforated ulcers are located in the duodenum. Perforated ulcer remains a serious condition with high morbidity and mortality [1]. The population affected by peptic ulcer perforation has changed during history. Today most of the patients show increasing age and co-morbidity, often resulting in higher mortality. Perforated peptic ulcer in the elderly with high co-morbidity is a high-risk condition [2, 3].

Since the late 1800s, the traditional treatment of perforated peptic ulcer is surgery [4].

Johan Mikulicz-Radecki (1850–1905) was the first to describe surgical closure of a perforated peptic ulcer in 1885 [5]. This procedure can now be performed using open or laparoscopic surgical techniques but still carries high morbidity (35%) and mortality (5–16%) [6].

Patients with high surgical risks have been treated conservatively with nasogastric tube and suction, also called Taylor’s method [7]. According to Alizadeh et al., conservatively treated patients have high mortality. In his retrospective study of 332 patients with perforated ulcer, 12 were treated conservatively with naso-gastric tube and antibiotics, and 8 out of these 12 patients died [8]. In a more recent study, conservative treatment was accompanied by a percutaneous drainage, and mortality was reduced to 20% [9]

Different flexible endoscopic methods have been used such as standard endoscopic clips in various ways and omental patches endoscopically pulled into the perforation. However, these techniques are only described as case reports [10].

Minimally invasive treatment of a perforated gastric ulcer with “over the scope clip” has been described in a case report [11]. This method is difficult to use in the case of a perforated duodenal ulcer due to the reduced space in the duodenum.

Other minimal invasive sewing techniques for flexible endoscopy, for example, with T-tags have been used to close anastomotic leakage and a perforated duodenal ulcer [12]. Unfortunately, the T-tags are not commercially available today.

Esophageal perforations have since the late 90-ies been treated with endoscopically placed covered stents and drainage of the pleura with very good results [13, 14]. Anastomotic leakage after gastric by-pass surgery has also been treated with the same method, covered metal stent and drainage, with good results [15, 16]. With this minimal invasive treatment, extensive surgery can be avoided, and early oral intake will be possible. Early oral intake has been shown to reduce post-operative mortality due to reduced bacterial translocation from the gut mucous membrane to the bloodstream [17]. Inspired by these findings, we started treating patients presenting with a perforated duodenal ulcer together with high co-morbidity or poor surgical candidates, with a partially covered stent and abdominal drainage. A case series of 8 patients treated between 2009 and 2012, presenting promising results, was published in 2013 [18]. To further investigate this new treatment strategy, we planned a randomized prospective study comparing stent treatment with surgical closure of perforate duodenal ulcers. The aim of this study was to investigate the safety and efficacy of the new treatment method compared with traditional surgical closure. This paper presents an intermediate analysis of data, according to the set protocol.

## Methods

### Power calculation

There are no published data that can be used for a power calculation of a study comparing endoscopic and surgical treatment techniques.

We performed a retrospective study at our own hospital including all patients treated for a perforated duodenal ulcer during 2009–2012. A total of 27 patients were identified, 19 were operated with surgical closure or resection, and 8 received stent treatment. In the surgically treated group, 8/19 (42%) patients had a complication compared with 2/8 (25%) in the stent group, showing a tendency towards fewer complications in the stent group, however, without statistical significance as the number of patients was limited.

To show non-difference in outcome after stent treatment or surgery, we assume that the new treatment (stent) results in 10% complications and that surgical closure results in 30%. Calculations give that 50 patients in each group will be needed to achieve 80% power with an a-level of 5%. An intermediate analysis will be performed when 50% of the inclusions are completed.

### Design

A multicenter randomized controlled trial was initiated at five regional hospitals in the Region of Västra Götaland, Sweden, to increase the number of included patients. All patients presenting at the ER with abdominal pain, clinical signs of a perforation of the upper gastrointestinal tract, and free abdominal air on a CT scan were approached for inclusion. Information about the study and informed consent was achieved by the surgeon on call. Inclusion took place between December 2014 and August 2018. Non-surgical candidates or patients in critical condition unable to sign the consent were not included. Patients under 18 years and patients in need of a translator were not approached for inclusion.

Randomization between surgical closure and stent treatment was performed after inclusion. Randomization was done by allocation of patients in a 1:1 ratio in balanced blocks. Envelopes were prepared with slips of paper marked with either surgery or stent, in blocks of six (three of each). Four envelopes were used out of each block.

Demographic data, ASA score, operation time, complications according to the Clavien-Dindo grading system [19], and hospital stay were recorded. Blood levels of CRP and WBC were followed at least 3 days post-operatively.

### Interventions and follow-up

Laparoscopy was performed in all patients to establish the diagnosis and to perform lavage. If needed, a peroperative gastroscopy was done to verify the presence of a perforated duodenal ulcer. Patients were then treated according to the assigned group. Surgical closure was performed with open or laparoscopic techniques according to the surgeon’s preference. Gastrostomy was avoided. In patients randomized to stent treatment, a per-operative gastroscopy was performed using a therapeutic gastroscope (Model GIF-2TH180; Olympus Corporation, Tokyo, Japan), allowing through the scope stent placement. The scope was passed beyond the point of perforation, a guide wire was placed through the scope into the proximal part of the jejunum, and a partially covered duodenal stent (Hanaro, MI-tech Korea) was advanced and released over the wire (Jagwire; Boston Scientific, Marlborough, M, USA) to cover the perforation. Care was taken to place the oral end of the stent above the pylorus and the covered part of the stent at the perforation site.

Abdominal lavage, using warm saline, was performed in all patients, and an abdominal passive 20 Fr drain was placed at the site of the perforation. All patients received broad spectrum antibiotics (Piperacillin-Tazobactam 4 g/0.5 g three times daily) and were treated with intravenous proton pump inhibitors (Pantoprazol 40 mg twice daily) until oral intake was possible.

During post-operative day one, a methylene blue test was performed in all patients (250 ml water mixed with 5 ml methylene blue given orally). If blue color was observed in the abdominal drain, the patient was further evaluated for a salvation stent treatment in the case of surgical closure, or new stent placement in the case of previous stent treatment.

If no sign of leakage was observed, the patient was allowed oral intake of liquids during the first post-operative day, increasing to soft food after a couple of days for the surgical group. To decrease the risk of stent migration, patients in the stent group were only allowed liquid diet until stent removal. Post-operative oral intake and nutrition were monitored by a nutritionist, and the daily need of calories was calculated for each patient in both groups. Supplementary parenteral nutrition was given if needed, in both groups. Liquid diet was adjusted to be as nutritious as ordinary diet.

Any complication was treated according to local guide lines.

Stents were endoscopically removed 2–3 weeks after placement, and the site of perforation was inspected. If there was any sign of remaining perforation, a new stent was placed for two more weeks.

### Statistics

Values are given as median and range. Comparisons between groups were performed using non-parametric tests, Wilcoxon signed rank test for related data, and the Mann–Whitney *U* test for non-related data and the *χ*^2^ test for nominal data. The Kruskal–Wallis test was used for multiple comparisons. All statistics were processed using the IBM SPSS 26 statistics software. Differences were considered statistically significant at *p* < 0.05.

## Results

Patients were included in only two of the 5 hospitals that initially intended to participate, mainly due to lack of experienced endoscopists on call for stent placement outside office hours.

**Fig. 1:**
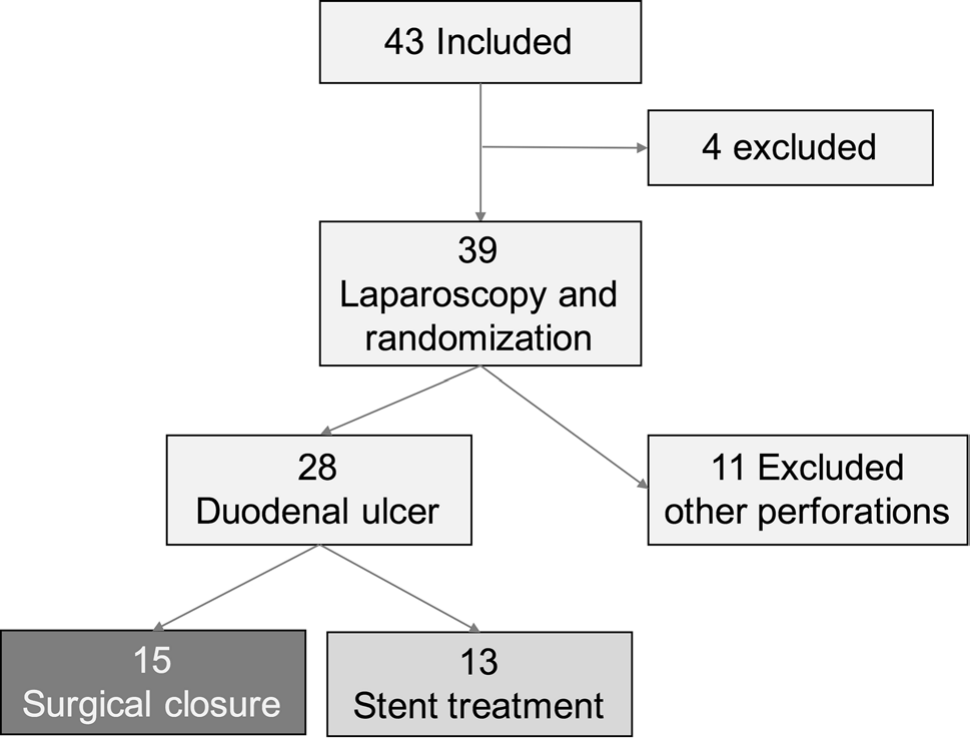
Inclusions and exclusions, 43 patients were included, 15 were excluded, 28 had a perforated duodenal ulcer, 15 were randomized to surgical closure, and 13 to stent treatment

A total of 43 patients were included in the study, one patient was excluded due to acute deterioration after inclusion and was assessed as a non-surgical candidate, and one patient withdrew the consent. 41 patients had a diagnostic laparoscopy, 13 of them were excluded: 10 had other perforations (gastric or colonic perforations), one patient had no visible perforation but free abdominal air and fluid in the abdominal cavity, and two patients were excluded due to protocol violation (Table 1). The remaining 28 patients had a confirmed perforated duodenal ulcer, 15 were randomized to surgical closure, and 13 to stent treatment (Fig. 1). In the surgical closure, group 5 had a laparoscopic closure and 10 were converted to open surgery for ulcer closure.

**Table 1:** Excluded patients

Excluded patients, total	15
Acute deterioration	1
Withdrawn consent	1
Colonic perforation	5
Gastric perforation	5
No perforation identified	1
Protocol violation	2

Overall median age was 77 years (23–91) with no difference between the treatment groups. Median age in the surgical group was 75 years (23–91) vs 80 years (38–87) in the stent treatment group (Table 2). A total of 15 women and 13 men were randomized, with no gender difference between the treatment groups (Table 2). Median age seemed to be slightly higher among the included women (82 years (37–89) than among the men (74 years (23–91)), but without statistical significance.

**Table 2:** Demographic data for the 28 included patients

Demographic data	Surgical closure	Stent treatment	All patients	
Number	15	13	28	
Age, years median (range)	75 years (23–91)	80 years (_._38–87)	77 years (23–91)	n.s
Gender, female/male	8 F/7 M	7 F/6 M	15 F/13 M	n.s
BMI, kg/m^2^ median (range)	28 (21–30)	24 (19–30)	27 (19–30)	n.s

**Fig. 2:**
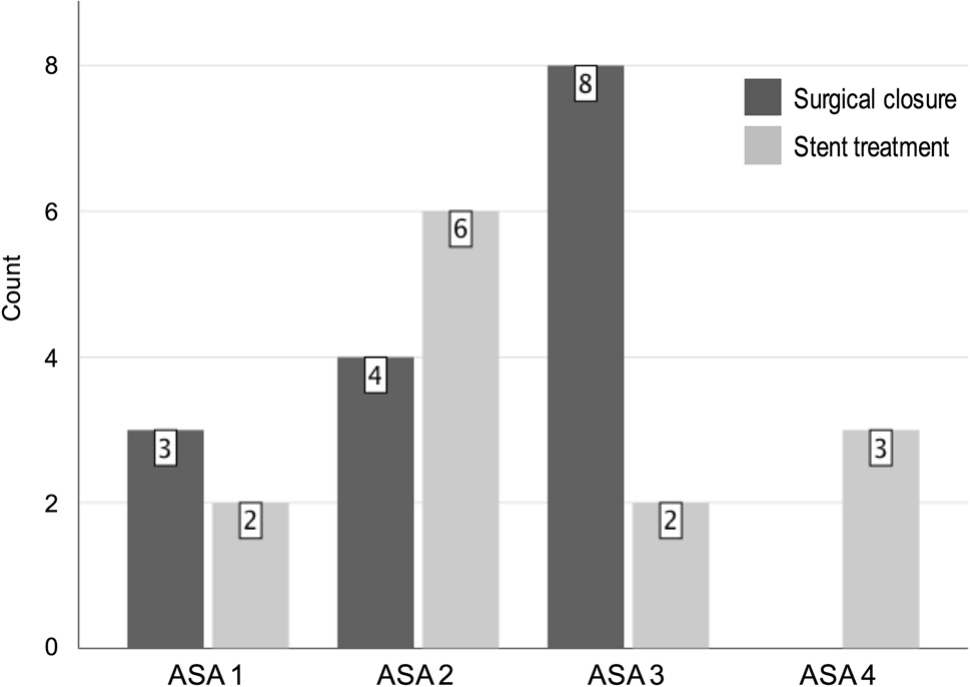
Number of patients with different ASA scores presented by treatment group. ASA score showed a tendency towards uneven distribution, comparing the two groups, *p* = 0.069 (*χ*^2^ test)

**Fig. 3:**
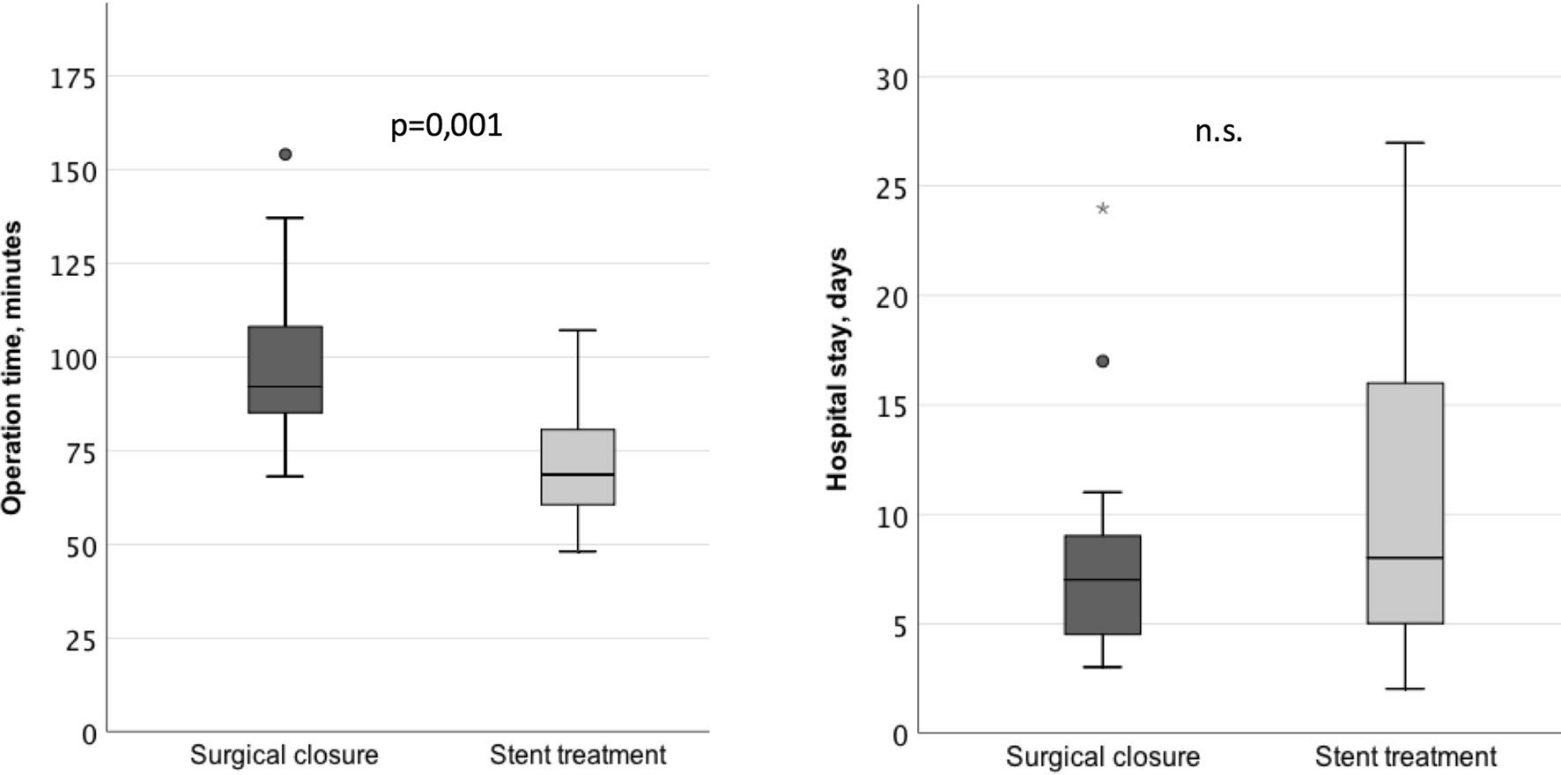
Operation time and hospital stay by the two treatment groups. Operation time was significantly shorter in the stent group, *p* = 0.001

**Fig. 4:**
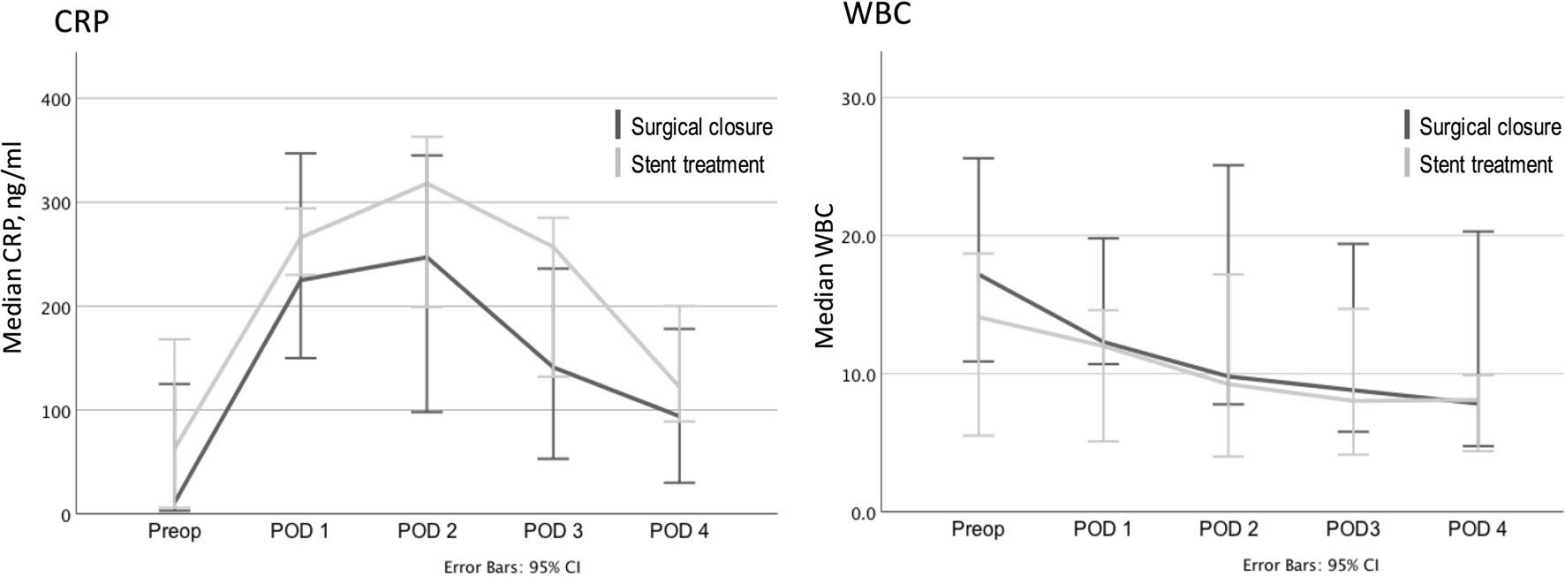
C-reactive protein (CRP) and White blood cell Count (WBC) before intervention and on post-operative days 1–4. Both groups showed a significant rise in CRP on POD 1 but no differences between the two treatment groups were found

ASA score showed a tendency towards uneven distribution comparing the two groups, 1–3 in the surgical group and 1–4 in the stent group. The three ASA 4 patients were all randomized to stent treatment (*p* = 0,069) (Fig. 2). In the surgical group, 5/15 were operated more than 12 h after symptom onset compared with 7/13 in the stent group (n.s.). Surgical closure was performed using laparoscopic technique in 5/15 patients, and 10/15 were converted to open surgery after the initial diagnostic laparoscopy. Operation time was significantly shorter in the stent group, 68 min (48–107) in comparison with the surgical closure group, and 92 min (68–154) (Fig. 3). Post-operative follow-up of CRP and WBC showed no significant differences between the groups (Fig. 4). All patients had a significant rise in CRP on post-op day 1 as expected. There was no difference between the groups regarding hospital stay and median stay was 7 days (3–24) in the surgery group vs 8 days (2–27) in the stent group (Fig. 3). Stents were removed after a median of 21 days (11–37 days) without complications.

**Fig. 5:**
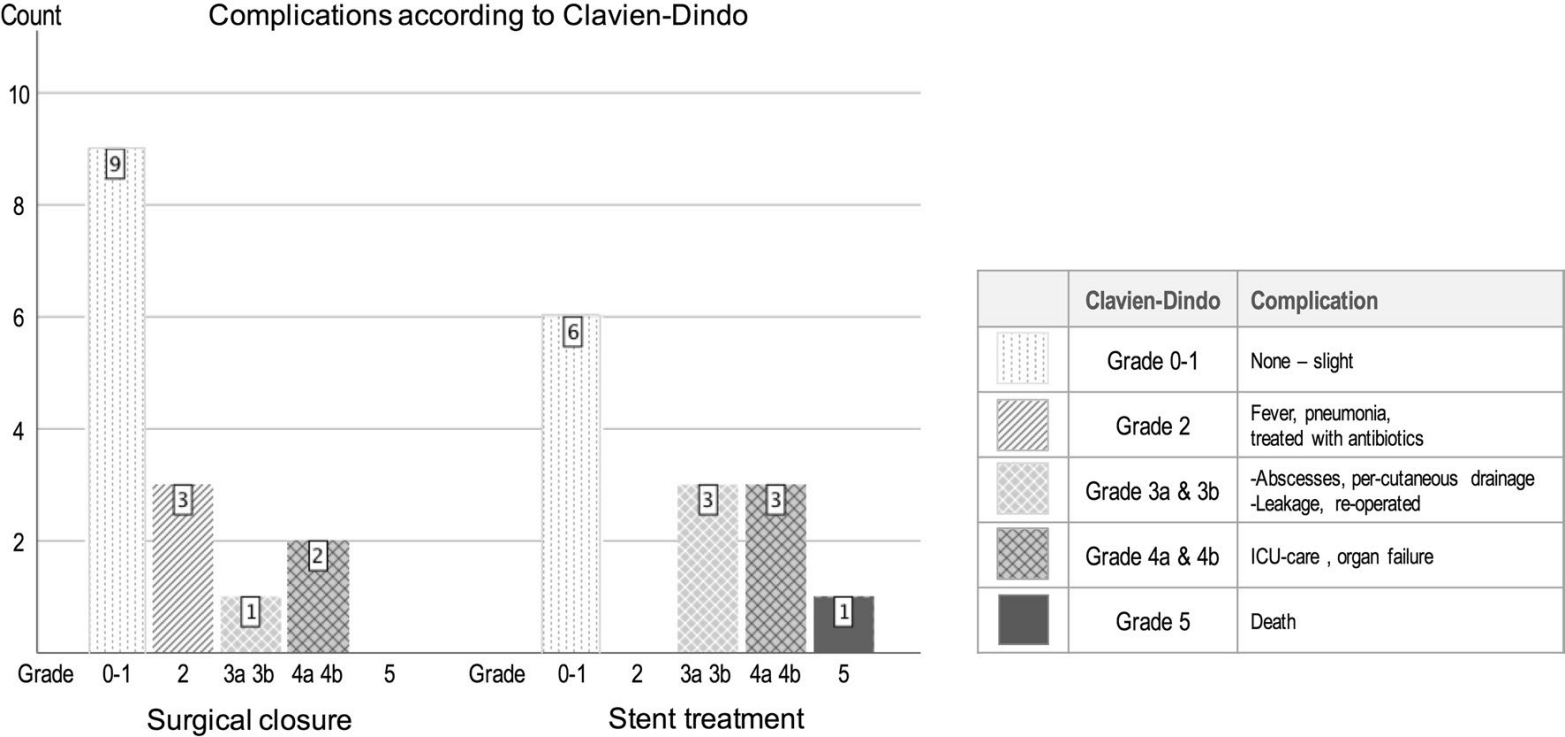
Distribution of complications graded by Clavien-Dindo for the two treatment groups. No statistical differences were found

Overall morbidity rate was 12/28 (42%) (Clavien-Dindo grade 2–4), and the mortality rate was 1/28 (4%). There was no significant difference in complication rates, Clavien-Dindo (C-D) grade 2–4, between the groups. Six patients in the surgical closure group had a complication (C-D 2–4), six patients in the stent group had a complication (C-D 2–4), and one patient died (C-D 5), (Fig. 5).

In the *surgical closure group*, two patients had post-operative non-specific fever, and one patient had pneumonia (C-D 2). One patient presented leakage, positive blue dye test, on post-operative day 1 and was treated with stent placement during 22 days. The same patient also developed an abdominal abscess needing percutaneous drainage (C-D 3). Two patients needed post-operative ICU care due to renal and circulatory failure needing inotropic support (C-D 4). One of them also needed total parenteral nutrition (TPN), due to a post-operative stricture at the surgical closure site. The stricture was not treated because of high age and comorbidity, and the patient died one month later in a nursing home.

In the *stent group*, two patients developed an abdominal abscess and were both treated with percutaneous drainage (C-D 3). One patient had signs of leakage, positive blue dye test, after stent placement and was treated with a new stent with good outcome. This patient also experienced dysphagia for a longer period due to a synchronous cancer of the ear (C-D 3). Three patients needed post-operative ICU care, circulatory failure in 1 case, and the combination of renal and circulatory failure in two cases. All of them were treated with inotropic support (C-D 4). One patient who preoperatively was in a deteriorated clinical condition developed post-operative multi-organ failure and died (C-D 5). This patient presented at the ER with a week-long history of abdominal pain, in a clinically septic condition, post-operatively developing atrial fibrillation and cardiac failure.

**Fig. 6:**
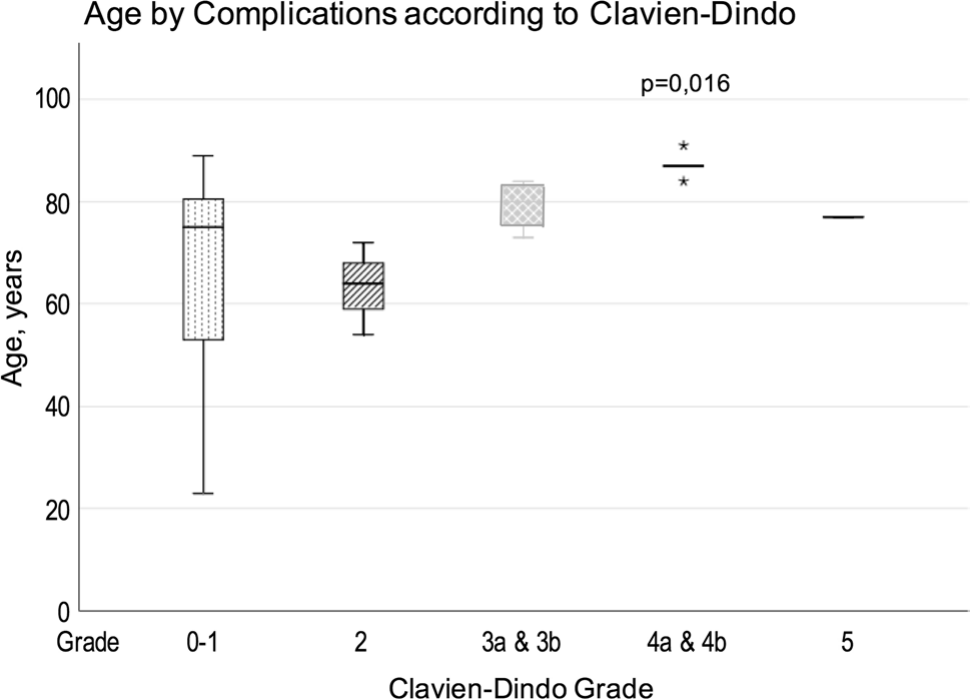
Age of all patients, presented by grade of complication. Patients with a grade 3–5 complication were significantly older than those without a complication or those with a grade 2 complication

The 10 patients who had a complication of Clavien-Dindo grade 3–4-5 were significantly older than those without a complication or those with a C-D 2 complication, median age in this group was 84 years (73–91) (*p* = 0.016) (Fig. 6).

**Fig. 7:**
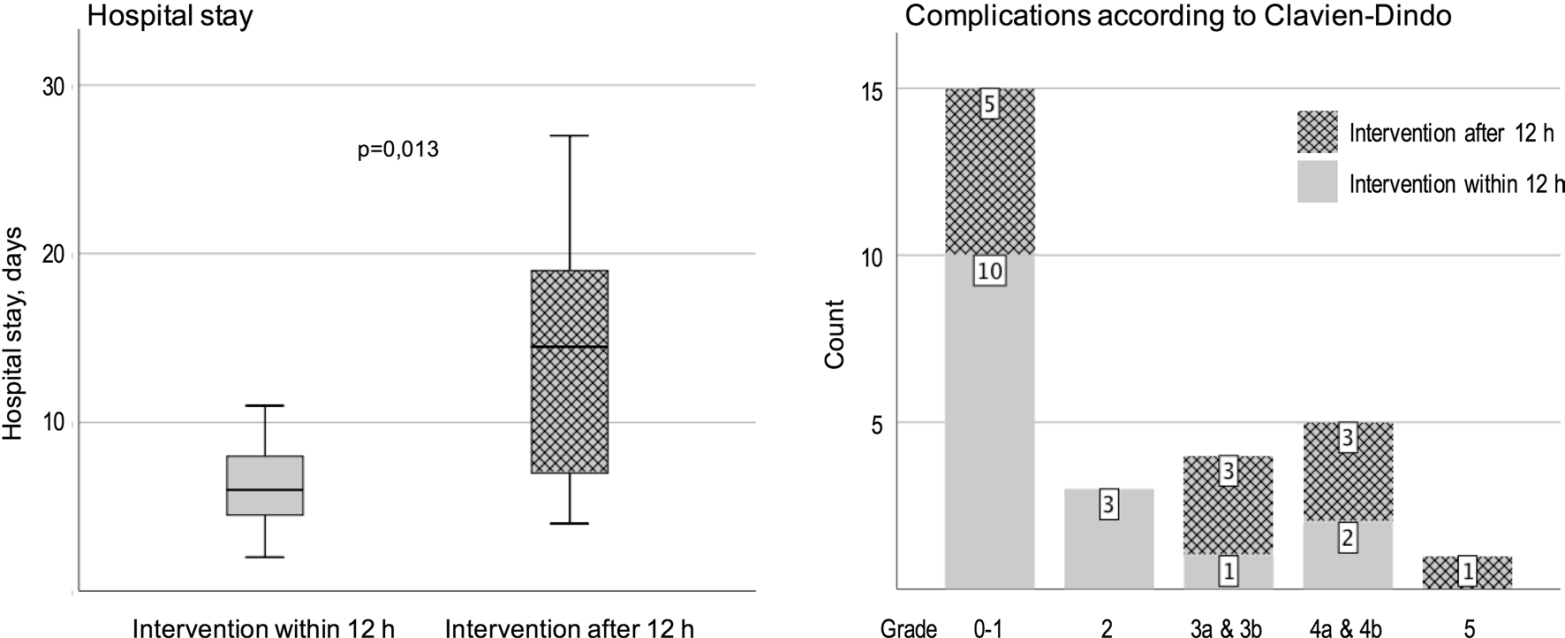
Hospital stay and distribution of complications among all patients, comparing intervention within 12 h and after 12 h since symptom onset. Patients who were treated after more than 12 h showed longer hospital stay and had more Clavien-Dindo grade 3–5 complications

Time to surgical intervention longer than 12 h from symptom onset showed a statistical correlation with the incidence of a grade 3–5 complication (*p* = 0.04). Out of the 4 patients with a C-D 3 complication (abscess or leakage), 3 were operated more than 12 h after symptom onset. For C-D 4 complications (ICU care with 1–2 failing organs), 3/5 had a late intervention and the only patient who died also had a late intervention (Fig. 7).

Patients who were operated more than 12 h after symptom onset had a longer hospital stay than those operated within 12 h (*p* < 0.013). Median stay was 13 days (4–27) for those treated after more than 12 h, vs 6 days (2–11) for those treated within 12 h (Fig. 7). This finding was the same for both treatment groups.

Patients with a complication (C-D 2–5) had significantly longer hospital stay than those without (*p* = 0.001). Median stay was 15 days (6–27) for patients with a complication vs 5 days (2–8) for those without. Surgical treatment or stenting did not affect this difference.

## Discussion

Our intermediate results, keeping the small sample size in mind, show no significant difference regarding morbidity or mortality after stent treatment or surgery for perforated duodenal ulcer. High age, delayed intervention, co-morbidity, smoking, and septic shock on arrival are all factors that increase both morbidity and mortality after a perforated duodenal ulcer, as reviewed by many authors [2, 3, 6]. The complication rate, Clavien-Dindo 2–4, in the current study was 12/28 patients (42%), which is consistent with reports in the literature [20, 21]. Late intervention, more than 12 h after symptom onset, was associated with a Clavien-Dindo grade 3–4 complication. Patients with these complications were also of high age, median 84 years (73–91). The 5 patients with a C-D 4 complications were all very old, with a median age of 87 years (84–91). They needed a median of 1 (1–4) post-operative days in the ICU with inotropic support to improve renal function. Interestingly, they did not have a high ASA score, ASA score was 2 (*n* = 3) or 3 (*n* = 2) on arrival. Their risk factor was age, and in 3/5 high serum Creatinine at admission, they all survived without persistent renal failure and left hospital in good shape. One patient died (1/28), which seems to be a low mortality rate compared with other studies [20, 21]. Boey showed, in a prospective study of 250 patients with perforated duodenal ulcers, that major medical illness (ASA score 4 & 5) preoperative shock and delayed surgery were accurate predictors of mortality [22]. The patient who died in our study had all of these predictors, including a high serum Creatinine and CRP on arrival, and died after 17 days in the ICU. This death was not believed to be related to the assigned treatment but rather to medical conditions prior to intervention.

Post-operative abscesses needing intervention, Clavien-Dindo 3, were found in 3 patients. Two of them occurred in the stent group, one sub-diaphragmatic and one in the pouch of Douglas, locations that are difficult to lavage and clean laparoscopically. One abscess occurred in the surgical group, located at the site of ulcer perforation, following post-operative leakage. All patients had an abdominal drain placed at the site of perforation. The drains were not placed to prevent abscess formation but to show signs of leakage.

Post-operative leakage after surgical closure of a perforated duodenal ulcer occurs in 3–6% according to recent papers [6, 21, 23]. In the current study, leakage occurred in two patients (2/28), one after open closure and one after stent treatment. These patients had preoperative ASA scores of 3 and 4, respectively. Both leakages were treated by placement of a covered stent. In the patient who was stented as primary treatment, the first stent had slipped out of the pylorus into the bulb, allowing leakage. It was removed and replaced by a similar stent, better adjusted over the pylorus. In the case of primary surgical closure, a covered stent was placed over the site of leakage, in line with salvage treatments after suture leaks in, for example, bariatric surgery [16]. The first two patients in our case series from 2013 were treated the same way, with a covered stent to treat post-operative suture-line leakages after open surgical closure, both with good result [18].

Conservative treatment with nasogastric tube and suction together with antibiotics was introduced by Taylor during the 40s. He believed that spontaneous sealing of a perforated ulcer could occur in selected cases, and treated 28 consecutive patients using this method, whereof 4 died [7]. In 1989, Croft was the first to compare two different methods for treating ulcer perforations. He performed a randomized study comparing conservative treatment and surgical closure with surprisingly good results in both groups [24]. However, in both these studies, the diagnoses of ulcer perforation in non-operated patients were not verified. Criteria were clinical signs and symptoms of a viscus perforation together with free air on X-ray or later CT scan. In the current study, 1/4 of included patients had non-duodenal ulcer perforations, despite the same main inclusion criteria. It is reasonable to believe that this phenomenon also occurred in the above-mentioned studies, making conclusions on conservative treatment of ulcer perforation from those studies somewhat uncertain. Our study indicates that the diagnosis of a perforated ulcer cannot be established without either gastroscopy or laparoscopy. Croft concluded, in his study, that conservative treatment might not be a good option in elderly patients, who might be less prone to spontaneous ulcer sealing [24]. In our current study and in our previous case series [18], age did not seem to influence the clinical outcome of stent treatment, where the ulcer seals when the leak is covered.

During this study, all patients in the stent group were kept on liquid diet for the duration of the stent treatment, median 21 days. This regime was part of the study protocol and decided on to minimize the risk of stent migration. In patients outside the current study, treated for leakage with a partially covered stent, we have moved to allowing soft food after a couple of days with liquid diet. This routine seems reasonable as the ingrowth into the uncovered flares of the stent can be assumed to have started. So far no event of stent migration has been recorded.

Operation time was significantly longer in the surgical closure group. This group includes both open and laparoscopic procedures with a tendency to shorter operating time for the laparoscopic approach. Despite the small sample size, it seems that laparoscopic sutured closure and stent treatment together with laparoscopic lavage show similar operation times. Economically, stent treatment might therefore end up somewhat more expensive when compared to laparoscopic surgical closure. However, in cases where it is difficult to find the perforation, e.g., in obese patients or in patients with previous upper abdominal surgery, stent treatment might be cost effective.

A major limitation of this study is the small sample size. We decided to perform this intermediate analysis after nearly 4 years of inclusion, to verify safety. Inclusion of patients was demanding as it often took place out of office hours and the incidence of ulcer perforation is low and decreasing [1]. Another limitation is that only patients capable of understanding information and giving consent are included in our study, why the selection might be biased towards less co-morbid patients. Patients in preoperative shock are often in a confusional state and therefore not eligible for inclusion.

However, we believe that the results from this intermediate analysis are important despite its small sample size. Our main conclusion is that stent treatment together with laparoscopic lavage and drainage offers a safe alternative to traditional surgical closure in perforated duodenal ulcer. Stent treatment also seems to be a good alternative in cases of suture-line leakage. A larger sample size would be necessary to show non-inferiority regarding stent treatment.
